# Design of linear and cyclic peptide binders from protein sequence information

**DOI:** 10.1038/s42004-025-01601-3

**Published:** 2025-07-22

**Authors:** Qiuzhen Li, Efstathios Nikolaos Vlachos, Patrick Bryant

**Affiliations:** Science for Life Laboratory, The Department of Molecular Biosciences, The Wenner-Gren Institute, Stockholm University, Solna, 171 65 Sweden

**Keywords:** Protein design, Peptides, Computational chemistry, Structure prediction

## Abstract

Structure prediction technology has transformed protein design, yet key challenges remain, particularly in designing novel functions. Many proteins function through interactions with other proteins, making the rational design of these interactions a central problem. While most efforts focus on large, stable proteins, shorter peptides offer advantages such as lower manufacturing costs, reduced steric hindrance, and improved cell permeability when cyclised. However, their flexibility and limited structural data make them difficult to design. Here, we introduce EvoBind2, a method for designing novel linear and cyclic peptide binders of varying lengths using only the sequence of a target protein. Unlike existing approaches, EvoBind2 does not require prior knowledge of binding sites or predefined binder lengths, making it a fully blind design process. For one target protein, we demonstrate that linear and cyclic peptide binders of different lengths can be designed in a single shot, and adversarial designs can be avoided through orthogonal in silico evaluation.

## Abstract (web summary)

Shorter peptides are advantageous as protein binders, as they offer high-affinity binding to a diverse set of targets and lower manufacturing costs, but their flexibility and limited structural data make them difficult to design. Here, the authors introduce EvoBind2, a method for designing novel linear and cyclic peptide binders of varying lengths using only the sequence of a target protein, without prior knowledge of binding sites or predefined binder lengths.

## Introduction

Protein design is in its golden age due to the advent of accurate structure prediction technology^1–3^. While single protein structures can now be designed with high accuracy^4,5^, designing new functions, such as binding, without relying on structural information remains challenging^6,7^. Additionally, there are numerous issues to address before designed proteins can benefit society, including scalable production. However, shorter proteins, such as peptides, can bind with high affinity to a diverse set of targets^8,9^ and are cost-effective to produce via conventional chemical synthesis.

The success rate in predicting the structure of protein complexes using AlphaFold2 (AF) and AlphaFold-Multimer (AFM) is approximately 60%^2,3^. However, for protein-peptide complexes, AF reports a high false negative rate (84/96)^10,11^. While AF can identify true binders with high confidence, it misses most^12^. Therefore, the challenge in peptide binder design is to find a sequence likely to bind that AF can also accurately predict. The subset of such sequences among the 20 ^L^ possibilities may be very small (or even absent), necessitating a highly efficient and robust search process.

Currently, most protein design workflows follow a two-step process. Methods such as RFdiffusion^5^ and backpropagation through AF^13^ are used to generate backbone scaffolds for both single- and multi-chain proteins, after which the sequences for these scaffolds are redesigned using ProteinMPNN^14^. This inverse folding approach has been applied to binder design^14,15^, however, it relies on predefined scaffolds, which are unavailable for novel targets, and has so far been limited to designing larger proteins rather than peptides^6,7,16^. While recent methods have been developed to predict and design peptide structures^17^, they do not account for interactions with a target protein, and generating sequences for these peptides remains an open challenge. Moreover, only 4-7% of structural seeds can be successfully redesigned into sequences that structure prediction networks recognise using inverse folding^10^, highlighting the limitations of current approaches.

Alternatively, a joint search over a relaxed sequence-structure space^18,19^ enables the simultaneous design of both sequence and structure. This approach increases the likelihood of success by exploring possible sequence-structure combinations directly. Some binder design methods^19^ leverage this relaxed space using the loss function from our previously developed EvoBind (v1)^18^. However, no existing approach is fully untargeted and current methods require either a predefined binding site or a target scaffold. Additionally, no method to date can design cyclic peptide binders, which could offer advantages such as extended half-life and potential oral bioavailability ^20^.

AF relies on coevolution to produce accurate predictions and has been trained to be robust to minor changes in a multiple sequence alignment (MSA), thereby learning the redundancy between sequence and structure^21^. For design tasks, where no MSA or coevolution exists and only a single sequence is used^14,22^, AF must infer the structure from the single sequence. Small changes such as single mutations can now have a significant impact on the outcome and it has been shown that AF can distinguish such changes in the binding residues of peptides^10,19^.

Building on these principles, we developed a method to generate peptide binders using only the sequence of a target protein. Our approach allows AF to autonomously determine the binding site, binding sequence, and structure without predefined constraints. To mitigate adversarial designs and enhance success rates, we integrate a modified version of AFM^2^ for evaluation. Additionally, we introduce a cyclic offset^23,24^, demonstrating that cyclic peptide binders can achieve efficacy comparable to linear binders using the same input: a single protein target sequence.

## Results

### Linear and Cyclic peptide binder design

**Fig. 1:**
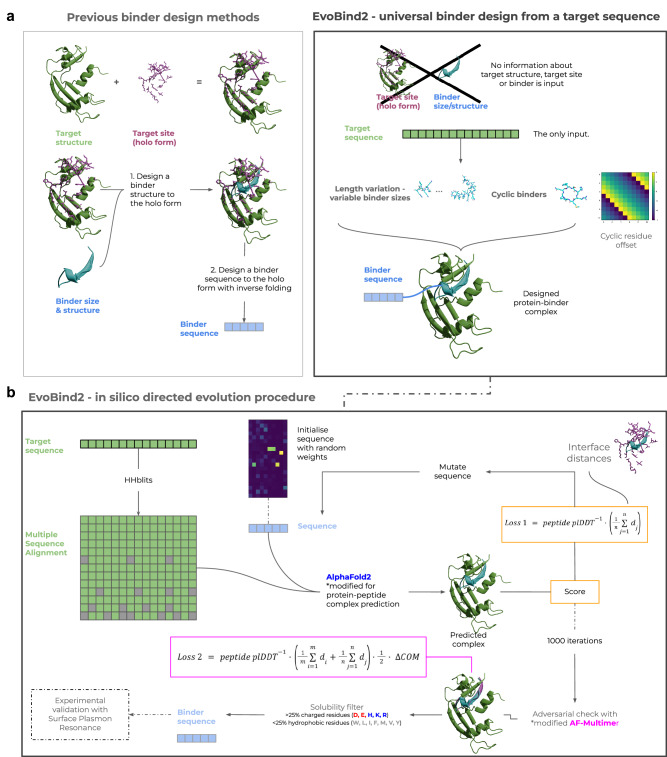
Description of EvoBind2. **a** Other methods for binder design rely on structural knowledge of target structures, binding sites in holo form and binders. This hampers the development of novel binders as binding information is required as input. In contrast, EvoBind2 is based solely on the sequence of the protein target. This makes EvoBind2 fully flexible and a binding sequence and protein-peptide complex structure is generated and evaluated simultaneously. The user can define the length (number of amino acids) of the binder and we find that Evobind can adapt the design to various peptide lengths (Fig. 2). By implementing a cyclic offset, we also enable cyclic peptide binder design offering potential cell membrane traversal and increased peptide half-lives. **b** Procedure for the design of peptide binders with EvoBind2. The target protein sequence is the only input to the design pipeline. No information about the binding site or peptide length is provided. The target sequence is searched with HHblits against Uniclust30, resulting in a multiple sequence alignment (MSA). The peptide sequence is randomly initialised with weights in the range 0–1 from a Gumbel distribution and the argmax at each position (Lx20) is used as the initial amino acid sequence. The MSA and the initial peptide sequence is input to a modified version of AlphaFold2 to predict the structure of the protein-peptide complex. A score (Loss 1) is calculated from the result. If the loss is lower than previously, the peptide sequence is used as a starting point for introducing a mutation and the complex prediction is then repeated. The sequence is subject to 1000 rounds of mutation and the AlphaFold2 predictions are then compared to predictions from a modified AlphaFold-multimer (Loss 2). This adversarial check is followed by a solubility filter and the final sequences are experimentally evaluated using surface plasmon resonance.

Many proteins exert their functions through interactions. The ability to target specific proteins through binding is essential for numerous biotechnological applications such as fluorescent labelling^25^, payload delivery^26^, targeted degradation^27^ and receptor activation/inhibition^28,29^. Current methods for protein binder design rely on knowledge of the target protein structure and binding site^14^ and even that of the binder^7^ (Fig. 1a). For novel targets, information about (1) target structure, (2) target site (residues), or (3) binder size is often unknown. In addition, most methods design large binders with well-defined structures, but shorter peptides are less sterically hindered and are cost-effective to produce via conventional chemical synthesis. Therefore, we set out to design novel peptide binders for a target protein using only the target protein sequence.

To design binders, we create a framework based on protein structure prediction^1^, EvoBind2, for in silico directed evolution^18^ (Fig. 1b). EvoBind2 iteratively mutates a peptide binder sequence to minimise the distance from the predicted positions of the peptide atoms towards any receptor protein atom (interface distances) and maximise the predicted confidence (peptide plDDT, predicted local distance difference test^1,30^, Loss 1, Eq. 1). This approach grants EvoBind2 complete freedom to generate an optimal sequence-structure combination for a peptide binder. By removing constraints, it eliminates procedural bias, ensuring that binding structures, sequences, and sites are not influenced by preconceived notions which may otherwise be suboptimal.

The target sequence is searched against Uniclust30 with HHblits, generating a multiple sequence alignment (MSA). The peptide sequence is randomly initialised, and the resulting sequence is fed into a modified AlphaFold2 (AF) to predict the protein-peptide complex structure. A score (Loss 1) is computed from the prediction. If this loss improves upon previous iterations, the updated peptide sequence serves as the new starting point for mutation, and the process is repeated. The sequence undergoes 1000 rounds of mutation, with each iteration evaluated using AF predictions. These predictions are then cross-validated with AlphaFold-Multimer (AFM) to compute a second loss score (Loss 2), ensuring an adversarial check. Following this, a solubility filter is applied, and the final sequences are experimentally validated using surface plasmon resonance (SPR).

**Fig. 2:**
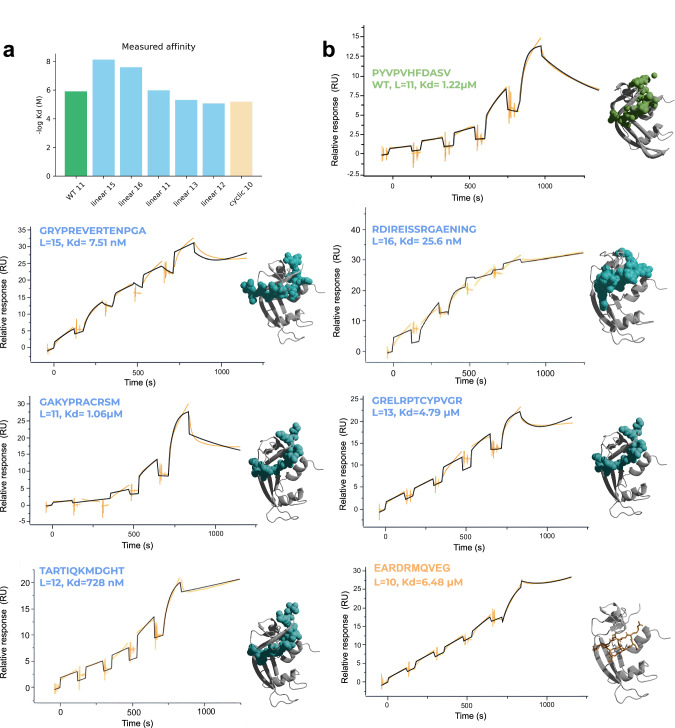
SPR results. **a** Barchart of the measured affinity (Kd) on a negative log scale for the WT and successful linear and cyclic designs. Each bar represents one sensorgram from Fig. 2b. **b** Single-cycle kinetics SPR sensorgrams and structure for the WT and successful linear and cyclic designs. The WT structure is the experimentally determined one from the PDB (PDB ID 1SSC: https://www.rcsb.org/structure/1ssc) and the designs are predicted structures from EvoBind2. The target protein (ribonuclease) is shown in grey and the peptide binder in orange stick format (cyclic) and cyan or green spheres (linear, WT). The SPR signal is outlined in orange and the fit used to determine the Kd in black. In the linear case, 5/13 of the evaluated binders have at least μM affinity and in the cyclic case, 1/4. The strongest linear binder has a Kd 162 times lower than the WT (7.5 nM vs 1.2 μM) and the cyclic is almost on par with the WT (6.48 μM).

Using EvoBind2, we designed both linear and cyclic peptide binders of 8-20 residues targeting the semi-synthetic Ribonuclease (PDB ID 1SSC: https://www.rcsb.org/structure/1ssc). We expressed and purified the protein as a construct together with the MBP fusion protein, which was included in downstream analyses, synthesised the peptides and measured the binding affinity of the designed peptides using SPR. We selected the top sequence for each length (*n *= 13 lengths) in the linear case and the top 5 lengths in the cyclic one, although one of these could not be easily synthesised (Methods). We found that 5 out of 13 (38%) of the designed linear peptide binders displayed μM affinity, with the strongest binder having a dissociation constant (Kd) of 7.5 nM (Fig. 2).

For the cyclic designs, we evaluated the top four designs experimentally, and length 10 obtained a Kd in the μM range (6.5 μM) (25% success rate, Fig. 2). Compared to the positive control (Kd = 1.2 μM), the strongest linear binder has 162 times the affinity and the cyclic is almost on par with the positive control, from a single selected sequence per length. Additionally, the peptides display a diverse set of sequences and predicted binding modalities, with the highest number of matching amino acids in contact with the WT being 21/65 (32%) across all successful designs (Supplementary Fig. 1). This highlights EvoBind2’s capability to generate varied and novel solutions.

We note that neither AF nor AFM have seen any structures similar to the designed protein-peptide complexes (especially in the cyclic case), suggesting the capability of EvoBind2 to generalise to unseen targets. The loss function used for the optimisation is defined as:

1 $${Loss}1={{peptide\; plDDT}}^{-1}\cdot \left(\frac{1}{n}{\sum }_{j=1}^{n}{d}_{j}\right)$$

Where the peptide plDDT (the predicted local distance difference test^30^) is the average plDDT over the peptide and dj is the shortest distance between all atoms n in the peptide and any atom in the target protein. The plDDT evaluates the local confidence of predicted protein structures. Higher plDDT scores indicate higher confidence.

### Adversarial designs

When designing binders using AF, it is possible to generate sequences that achieve high in silico confidence (low loss, Eq. 1) but fail to bind in reality. These deceptive sequences, which “trick” EvoBind2, are considered adversarial designs^31^. To mitigate this, we modified AFM^2^ to predict protein-ligand complexes using only MSA information for the receptor protein and a single sequence for the peptide. This additional validation step enhances design reliability, as it is unlikely that two independently trained structure prediction networks, using different data, would both agree on an incorrect solution.

**Fig. 3:**
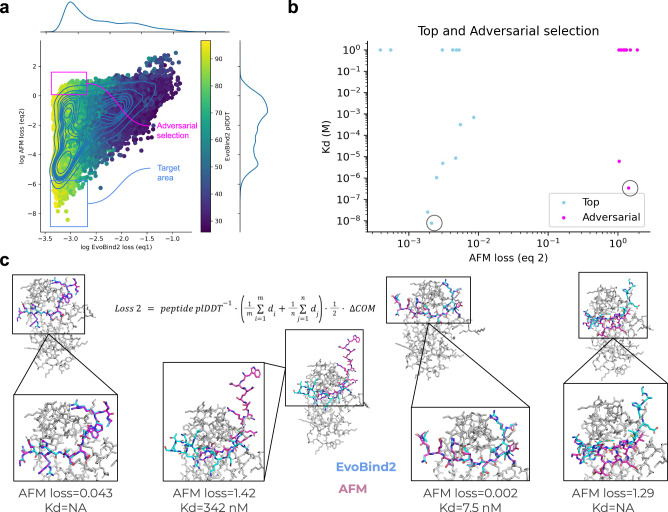
Adversarial validation. **a** EvoBind2 loss (Eq. 1) vs AlphaFold-multimer (AFM) loss (Eq. 2) on log scale for all linear design runs (*n *= 64935). The points are coloured by the plDDT from EvoBind2 and the density is represented with blue lines. The target area represents cases where both losses are low, and the adversarial selection cases where the EvoBind2 loss is low but the AFM loss is high. The discrepancy between the losses in the adversarial area signifies that those sequences may trick EvoBind2. **b** Affinity (Kd) vs AFM loss (eq2) for both the top selection (in the target area, Fig. 3a) and the adversarial selection (AFM loss>1) for the linear design (*n *= 13). The peptides with no detectable affinity are set to 1 M. The peptides with low AFM loss have lower Kd overall (6/13 with μM affinity) compared to sequences with AFM loss>1 (2/13 with μM affinity). The circled points mark examples shown in (**c**). **c** Examples of adversarial selections. The predicted structure is shown in stick format with the receptor structures in grey and the predicted peptide structures in cyan/magenta for EvoBind2/AlphaFold-multimer (AFM), respectively. The AFM loss (Loss 2) is reported together with the Kd measured by SPR (See Supplementary Fig. 2 for all sensorgrams). Left; an example adversarial to AFM (length=14) where the Kd is 342 nM although the AFM loss is high and undetectable at a low AFM loss. Right; an example where the adversarial prediction of AFM is accurate (length=15) and the Kd is 7.5 nM with a low AFM loss and undetectable at a high AFM loss.

To assess the effectiveness of the adversarial check, we selected sequences from the linear design where EvoBind2 and AFM disagreed. Specifically, cases where the AFM loss (Eq. 2) was high, but the EvoBind2 loss (Eq. 1) was low (Fig. 3). For each peptide length, we identified the sequence with the lowest EvoBind2 loss while maintaining an AFM loss >1, resulting in EvoBind2 losses ranging from 0.04 to 0.1. These peptides were then synthesized and experimentally evaluated for binding affinity against the target protein using SPR.

We found that only two of the sequences in the adversarial selection displayed μM affinity compared to five when both losses (Eqs. 1 and 2) were low. This suggests that the adversarial check effectively distinguishes true binders from false positives, improving the success rate by 2.5x. However, some sequences may be adversarial to AFM, as 2 out of 13 still display affinity in the μM range (Fig. 3c).

The loss for the adversarial selection is defined as:

2 $${Loss}\cdot 2={{peptide\; plDDT}}^{-1}\cdot \left(\frac{1}{m}{\sum }_{i=1}^{m}{d}_{i}+\frac{1}{n}{\sum }_{j=1}^{n}{d}_{j}\right)\cdot \frac{1}{2}\cdot \varDelta {COM}$$

Where the peptide plDDT is the average plDDT over the peptide, di is the shortest distance between all atoms in the receptor target atoms (Cβs within 8 Å between the peptide and the receptor protein as predicted by EvoBind2) m and any atom in the peptide, dj is the shortest distance between all atoms n in the peptide and any atom in the receptor target atoms and ΔCOM is the CA centre of mass distance between the predicted peptides from the design and validation procedures, respectively.

### Affinity and in silico metrics

**Fig. 4:**
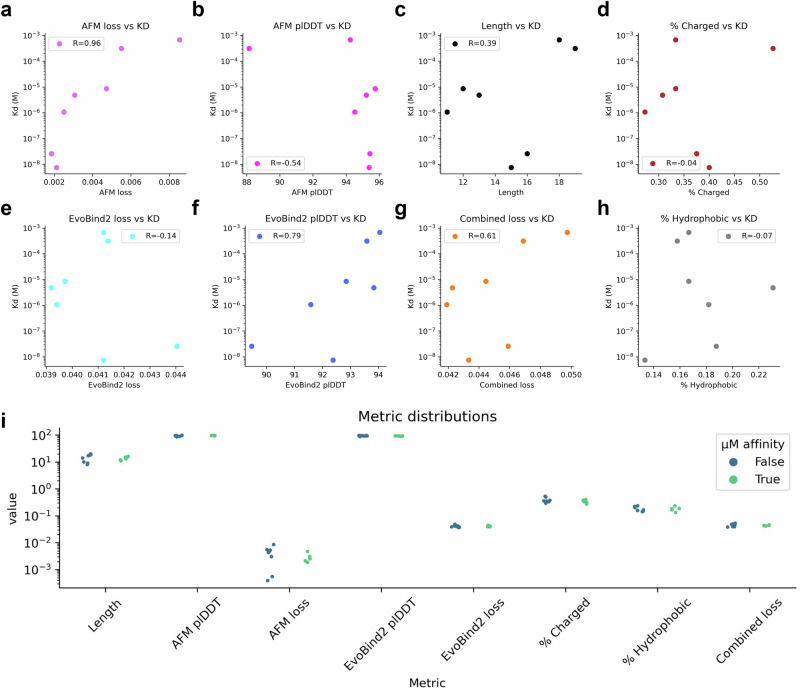
Affinity and metrics. **a**–**h** Scatter plots showing the affinities of linear peptides (KD) vs in silico metrics for designs with measurable affinity from the top selection (*n *= 7). The Spearman correlation coefficient (R) is annotated in each subfigure. Both the AFM loss and the EvoBind2 plDDT display a meaningful correlation with the measured Kd (*R *= 0.96 and 0.79, respectively). **a** AlphaFold-multimer loss (Eq. 2), **b** plDDT from AlphaFold-multimer, **c** the length of designed peptide, **d** the fraction of charged amino acids in the sequence, **e** plDDT from EvoBind2, **f** the loss from EvoBind2 (Eq. 1), **g** the combined loss from EvoBind2 and AlphaFold-multimer (Eqs. 1 and 2), and **h** the fraction of hydrophobic amino acids in the sequence. **i** Strip plot of the categories in **a**–**h** divided by if μM affinity could be measured/not (*n *= 13). No in silico metric separates true from false binders.

The loss functions (Eqs. 1 and 2) used in this study produce high-affinity binders, but not all selected binders display measurable affinity. To determine whether we can distinguish between true and false binders or predict affinity, we analysed various in silico metrics (Fig. 4). The best indicator for affinity is the AFM loss, with a Spearman correlation of 0.96. One of the most important metrics used in the loss functions is the plDDT^30^, which evaluates the per-residue confidence of predicted protein structures. Higher plDDT scores indicate higher confidence, and we observe a strong negative trend (higher plDDT, lower affinity) between the dissociation constant (Kd) and EvoBind2 (AF) plDDT scores (*R *= 0.79) and a positive one (higher plDDT, higher affinity) with the AFM plDDT (*R *= −0.54) (Fig. 4b, f). We also analysed the Predicted Aligned Error (PAE) as a complementary in silico metric analogous to the plDDT, but found that the Spearman correlation is lower (0.51, Supplementary Fig. 3).

The length of a peptide can influence its function, as longer peptides offer greater complexity and potential for diverse interactions. However, we find a weak relationship between peptide length and Kd (Fig. 4c), suggesting that the binding residues themselves are what matter most (Supplementary Fig. 1). A comparison of all metrics with the presence or absence of measured μM affinity is displayed in Fig. 4i, highlighting that in silico metrics fail to effectively distinguish between all true and false binders. This underlines the complexity of predicting protein-peptide interactions. We also included the adversarial selection and analysed all metrics in Fig. 4, reaching the same conclusions, although with lower Spearman correlations (Supplementary Fig. 4).

### Factors influencing the binder optimisation

**Fig. 5:**
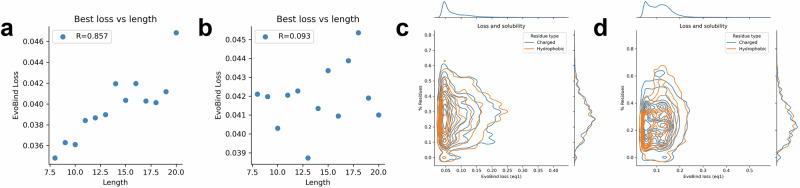
Optimisation metrics. Best EvoBind2 loss (Eq. 1) vs length for linear (**a**) and cyclic (**b**) design. For the linear design, the loss increases with the length (Spearman *R *= 0.857), but for the cyclic, such a relationship is not as apparent (Spearman *R *= 0.093). **c**, **d** Fraction of charged (D, K, R, H and E) and hydrophobic residues (W, L, I, F, M, V, Y) vs EvoBind2 loss (Eq. 1) for linear (**a**) and cyclic (**b**) design. Different fractions of charged/hydrophobic residues are observed for a variety of losses suggesting that the solubility is not important for EvoBind2 when generating designs.

Figure 5 presents the relationship between the best EvoBind2 loss (Eq. 1) and binder length for both linear and cyclic designs across five independent runs (initialisations). In the linear design, loss increases with length, whereas no such trend is observed for the cyclic design. While losses vary significantly between runs (Supplementary Figs. 5and 6), the results indicate that, given five attempts, comparable losses and affinities can be achieved for linear binders. The observation that longer sequences exhibit higher losses and greater variability may stem from the increased sequence-structure space, making it less likely to converge on an optimal design.

Beyond achieving high-affinity binders, peptides must also be functional under biological conditions to be suitable for biotechnological applications. To ensure this, we applied a solubility filter, requiring >25% of residues to be charged and <25% to be hydrophobic (Methods). However, these solubility metrics did not correlate with binding success (Fig. 5c, d) and could not be used to predict affinity (Fig. 4 d, h).

## Discussion

AI-based peptide binder design has the potential to rapidly expand the range of proteins that can be targeted for biotechnological applications. The EvoBind2 framework developed here designs peptide binders to a protein target using only its amino acid sequence, with no need for prior knowledge of binding sites, template structures, or binder sizes making it applicable to novel targets. We observed binding affinities (Kd) of 7.5 nM in the linear case and 6.5 μM in the cyclic compared to 1.2 μM for a known binder, achieving success rates of 38% and 25%, respectively. We note that binding was analysed with our target protein in an MBP-fusion construct, which may impact the SPR measurements, and is a limitation in the experimental evaluation here.

Unlike protein structure prediction, which relies on coevolutionary information from multiple sequence alignments (MSAs), de novo peptide design lacks this evolutionary context. The absence of coevolutionary information presents a significant challenge when designing new peptides, as the structure must be predicted without these constraints. The finding that successful peptide design is still possible^18^ suggests that important protein sequence-structure relationships have been learned independently of coevolutionary signals.

One of the key challenges in designing peptide binders is avoiding sequences that have high in silico confidence (plDDT) but fail to bind to the target protein, known as adversarial designs. To address this, we adapted AFM^2^ to predict protein-ligand complexes using MSA information for the receptor protein and a single sequence for the peptide. This additional validation step filters out sequences that appear promising according to EvoBind2 but do not function as true binders, enhancing the likelihood of successful linear binder design 2.5x. The rationale is that it is unlikely for two independently trained neural networks, based on different datasets, to agree on an incorrect prediction. This adversarial check distinguishes our method from previous design strategies that lacked such a validation step, which may explain their lower success rates^6,7,19^.

The protein targeted in this study, a semi-synthetic Ribonuclease (PDB ID 1SSC: https://www.rcsb.org/structure/1ssc) has a known peptide binder of length 11. To ensure the expressed protein is functional for affinity measurements, we synthesised this sequence and measured its affinity as a positive control, obtaining a Kd of 1.2 μM. Interestingly, the linear binders designed here outperform this control 162-fold, suggesting that EvoBind2 can not only generate new binders but also improve affinities for targets with known binders. While some of the SPR sensorgrams (e.g., for the length 16 peptide) exhibit a degree of noise, this does not affect the overall conclusions regarding EvoBind’s performance. High-affinity binding is still clearly observed, and the dose-response curves, particularly in comparison to the positive control in Fig. 1, would not be reproducible without genuine binding. We do, however, acknowledge the possibility that the presence of the fusion protein may have an impact here.

The predicted structures of the designed peptides show diverse binding modalities (Fig. 2, Supplementary Fig. 1). Using the AFM loss (Eq. 2) and plDDT from EvoBind2, it is also possible to regress the affinity with high accuracy (Fig. 4). We note that this analysis contains few data points and a single target. However, we can’t distinguish all true from non-binders, although sequences with a high AFM loss have a lower chance of binding. The findings highlight the importance of multi-modal validation and emphasise the need for wet-lab validation and careful interpretation when models disagree.

Cyclic peptides offer distinct advantages over linear peptides, including increased stability and potential for membrane traversal^32^, and several drugs on the market are cyclic peptides^33^. Interestingly, our results indicate that cyclic peptides can be designed to achieve comparable binding affinities using the same input information. In conclusion, our study demonstrates the potential of EvoBind2 for designing novel peptide binders with high affinity based solely on a target protein sequence. The fact that EvoBind2 does not require predefined binding sites or binder sizes is essential for targeting novel proteins, suggesting that this method could rapidly increase the number of proteins amenable to biotechnological applications. However, to fully assess the impact of EvoBind2 and protein design, additional targets and modalities will need to be validated.

## Methods

### Design protocol

#### EvoBind2

**Fig. 6:**
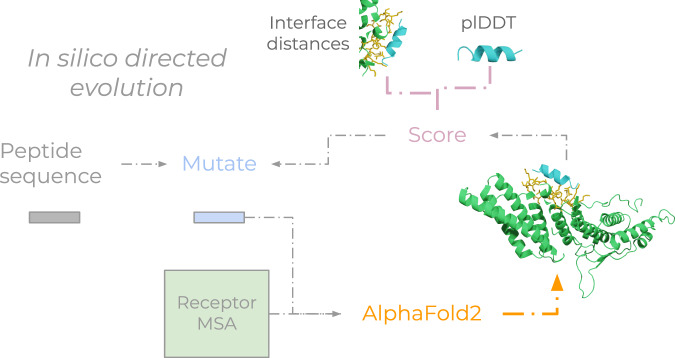
EvoBind2 overview. In silico directed evolution procedure towards an unspecified binding site. By not specifying a binding site, EvoBind2 is free to generate a structure-sequence combination to any part of the target protein as long as 1. the confidence (plDDT) of the predicted peptide is high and 2. the predicted peptide atom positions are close to the receptor surface (interface distances).

For designing peptide binders, we use a modified version of EvoBind^18^ (Fig. 6) that does not require a defined target area. This is the first method developed that can generate both sequence and structure from a single protein target sequence (to our knowledge). We design over a relaxed sequence-structure space that is searched by mutating one residue randomly at a time to minimise the loss function:

1 $${Loss}1={{peptide\; plDDT}}^{-1}\left(\frac{1}{n}{\sum }_{j=1}^{n}{d}_{j}\right)$$

Where the peptide plDDT is the average plDDT over the peptide and dj is the shortest distance between all atoms n in the peptide and any atom in the target receptor.

The receptor is represented by a multiple sequence alignment that is constructed from searching UniClust30_2018_08^34^ with HHblits^35^ version 3.1.0:


hhblits -E 0.001 -all -oa3m -n 2


The peptide is represented by a single sequence (initialised with a Gumbel distribution over all standard 20 amino acids). The single chain AlphaFold2 folding pipeline^1^ with model_1, one ensemble and 8 recycles was used. The optimisation is run for 1000 iterations and repeated five times from random starting points (sequences).

### In silico evaluation with AlphaFold-multimer to avoid adversarial designs

**Fig. 7:**
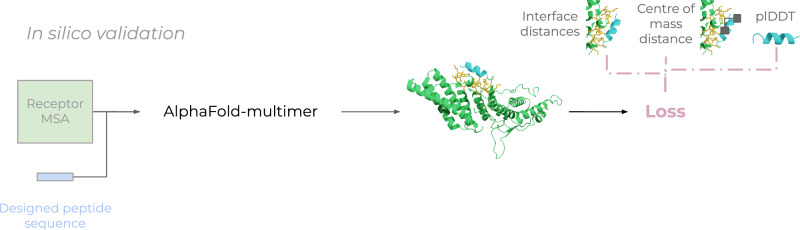
AFM validation. Given a receptor MSA and a single sequence for the designed peptide, AlphaFold-multimer is used to predict a protein-peptide complex. Based on the similarity of the predicted structure to that predicted with EvoBind2 and the confidence (plDDT) of the prediction a loss is calculated (Eq. 2).

In the EvoBind2 protocol developed here, no target site is used as this information is not available for novel target proteins. To ensure the design outcomes from the untargeted protocol are viable we use AlphaFold-multimer (AFM, Fig. 7) as an additional check to avoid adversarial designs. This allows us to utilise the original EvoBind1 loss function (Eq. 2) which relies on a specified target site and a known peptide binder. In this case, we compare the loss between the two predictions using the EvoBind2 designs as “pseudo-native” structures.

Previous benchmarking has found that the best option for protein-peptide complex prediction is AFM v2.1.0 (params_model_1_multimer_v2) with 3 recycles and dropout everywhere but in the structural model (highest correlation with the ranking confidence from AFM+median DockQ)^12^. To account for that some examples improve with more recycles, we set the number of recycles to 20 and include ‘early stopping’ (no more recycles are included if the confidence score is not improved).

Calculating the loss using EvoBind2 vs. AFM predictions circumvents the need to know the native structure or CA centre of mass to use the EvoBind loss function. The EvoBind loss function, which has demonstrated accuracy both in silico and in vitro^18,19^, can thus be used to evaluate the agreement between different neural networks.

2 $${Loss}2={{peptide\; plDDT}}^{-1}\cdot \left(\frac{1}{m}{\sum }_{i=1}^{m}{d}_{i}+\frac{1}{n}{\sum }_{j=1}^{n}{d}_{j}\right)\cdot \frac{1}{2}\cdot \varDelta {COM}$$

Where the peptide plDDT is the average plDDT from AFM over the peptide, di is the shortest distance between all atoms in the receptor target atoms (Cβs within 8 Å between the peptide and the receptor protein as predicted by EvoBind2) m and any atom in the peptide, dj is the shortest distance between all atoms n in the peptide and any atom in the receptor target atoms and ΔCOM is the CA centre of mass distance between the predicted peptides from the design and validation procedures, respectively. The di ensures the entire target region is covered as it is possible to get short peptide distances towards only a few of the target residues. If we don’t use both di and dj, similar scores could be obtained even though the predictions would differ substantially.

### Binder design selection

We select the designs with the best combined loss calculated as the sum of Eqs. 1 and 2. For the adversarial selection, we select designs with an AFM loss >1 according to Eq. 2 and the lowest possible loss according to Eq. 1, obtaining losses in the range 0.04-0.1 with Eq. 1 for the linear design task.

### Cyclic offset

To design cyclic binders, we implement a cyclic offset^23^, informing the structure prediction network to connect the peptide amino acids in a continuous cycle. This feature is called the relative positional encoding in the AlphaFold2^1^ and AlphaFold-multimer^2^ networks.

### PAE

The predicted aligned error (PAE) is a predicted confidence metric from the AlphaFold-multimer network that provides an estimate of the 2D distance error between residues^2^. Here, we extracted the PAE in the protein-peptide interface and averaged it to compare its relationship with the affinity (Kd) measured with SPR (Supplementary Fig. 3).

### Positive control

To ensure that the expressed protein (semi-synthetic Ribonuclease, PDB ID 1SSC: https://www.rcsb.org/structure/1ssc) is in a structural state where it can interact with designed binders we analysed the affinity of the known peptide binder (11 residues) with sequence: PYVPVHFDASV. Using single-cycle kinetics with surface plasmon resonance (SPR) we obtained a Kd of 1.2 μM (Fig. 2). At the same time, many of the adversarial designs report no response suggesting that the protein is in a selective structural state where only specific peptide sequences will bind with high affinity.

### Solubility

To ensure water solubility we select designed binders that have an average plDDT>90, >25% charged residues (D, K, R, H and E) and <25% hydrophobic residues (W, L, I, F, M, V, Y). Figure 6c and d show different fractions of charged/hydrophobic residues are observed for a variety of losses suggesting that solubility is not important for EvoBind2 when generating designs. All synthesised peptides were soluble in the SPR running buffer (see Affinity measurement with SPR).

### Contact similarity

To analyse the novelty of the designed peptides, we compare the AA contacts (Cβ < 8 Å) towards the receptor with the contacts from the known binder in the positive control. For the positive control, we use a crystallised structure and for the designs, the predicted structures from EvoBind2 (modified AlphaFold2). A visualisation of the amino acids in contact for each of the binders can be found in Supplementary Fig. 1. The number of contacts are 15, 10, 11, 7, 6 and 5 and the fractions of the number of WT contacts (65) are 0.23, 0.15, 0.32, 0.11, 0.09 and 0.8 for linear lengths 15, 16, 11, 13, 12 and cyclic 10, respectively.

### Protein expression and purification

The psfRNAseA-c001 construct was transformed into E. coli BL21 (DE3) T1R cells and cultivated in Terrific Broth (TB) medium supplemented with glycerol, ampicillin, and chloramphenicol. The cultures were grown at 37°C, and protein expression was induced with IPTG at an OD of approximately 3, after which the temperature was reduced to 18°C for overnight expression. The cells were harvested, resuspended in an IMAC lysis buffer, and disrupted by sonication. The soluble fractions were purified using IMAC and size exclusion chromatography, and the target protein was pooled, concentrated, and stored at -80°C. The psfRNAseA protein eluted together with some contaminants or possibly proteolytic fragments, and the full-length psfRNAseA protein with the MBP-8xHis tag intact was identified by SDS-PAGE analysis. Batch purity was analyzed using SDS-PAGE (see Supplementary Fig. 7for elution profiles and SDS-PAGE). For the SDS-PAGE, ~4 μg of protein was loaded. Protein concentration was calculated from UV absorbance at 280 nm using a theoretical extinction coefficient and the psfRNAseA-h001 (NS) fractions: B6 – B2 were pooled. While free MBP is present in the final protein mixture, it does not affect the SPR measurements, as the responses are normalised based on the amount of peptide added. In the worst-case scenario, if the MBP were removed entirely, the SPR response curves would likely be stronger, not weaker. Therefore, the presence of free MBP does not undermine the observed binding signals or the conclusions drawn from them.

### Peptide synthesis

Lyophilized powders of peptides designed by EvoBind2 were synthesised and purified by JPT Peptide Technologies GmbH (linear peptides) and GenScript (cyclic peptides). For the JPT synthesis, the net content purity is reported but for the cyclic peptides, the lyophilisation process may leave residual salts in the final product. While this does not affect the interpretation of the binding results, it is possible that such salt content could slightly suppress the observed affinities. Based on prior experience, we estimate that removal of these salts might result in an increase in apparent affinity by approximately 20–50%. Therefore, the reported affinities for cyclic peptides may be conservative estimates. For the top-selected cyclic peptide of length 17 (Supplementary Table 3), GenScript attempted several rounds of synthesis but was unable to produce the peptide. The quality of successfully synthesised peptides (purity above 90%, Supplementary Tables 1-3) was verified by high-performance liquid chromatography and mass spectrometry. Peptides were resuspended in HBS-P+ Buffer (0.01 M HEPES, 0.15 M NaCl, and 0.05% v/v Surfactant P20) to achieve a stock concentration of 10 mM.

### Affinity measurement with SPR

To directly measure the binding affinity of designed peptides toward 1SSC we used surface plasmon resonance (SPR). An experimental method that measures real-time binding interactions between biomolecules by detecting changes in refractive index near a sensor surface. All interaction analysis was performed at 25 °C in a running buffer of HBS-EP+ (10 mM HEPES, pH 7.4, 3 mM EDTA, 150 mM NaCl, 0.05% Tween-20) for Biacore 8 K. MBP-8xHis-1SSC was immobilised to about 12000 RU using standard amine coupling reagents 1-ethyl-3-(3-dimethylaminopropyl)carbodiimide (EDC) and N-hydroxysuccinimide (NHS) onto a Series S Sensor Chip CM5. The designed peptides were captured onto the prepared surface to generate an interaction with a maximum response (R_max_) from 8 RU to 35 RU (Fig. 2 and Supplementary Fig. 2).

Single-cycle kinetics experiments were conducted using different concentration ranges of the peptides as the analytes. A five-point concentration series was prepared through serial dilution based on peptide mass: 2 nM, 20 nM, 200 nM, 2 μM, and 20 μM. Each concentration series was injected in a single cycle without regeneration steps between injections. The flow rate was set at 30 μL/min, with an association time of 120 seconds and a dissociation time of 1800 seconds.

Data processing and analysis were performed using Biacore^TM^ Insight Evaluation Software. Raw data were preprocessed using both reference and blank subtraction to ensure data quality. For reference subtraction, we used a reference flow cell that was activated and deactivated without ligand immobilization to account for non-specific binding and bulk refractive index changes. Buffer blank injections were included before each concentration series, using the ‘Median consecutive preceding and following’ blank subtraction setting. Sensorgrams were then fitted to a 1:1 binding model (Eqs. 3 and 4)^36^ using global kinetic rate constants for association (ka) and dissociation (kd), along with R_max_ values per sample. The retained data were used to determine the association rate constant (ka), dissociation rate constant (kd), and equilibrium dissociation constant (KD).

3 $$A+B\leftrightarrow {AB}$$

4 $${Kd}=\frac{\left[A\right]\cdot \left[B\right]}{\left[{AB}\right]}=\frac{{kd}}{{ka}}$$

Where A is the protein receptor that was immobilised on the sensor chip, and B is the analyte (peptide). The equilibrium dissociation constant Kd is calculated as the ratio of kd to ka, where ka represents the association rate constant for complex formation (A + B → AB, M^-1^s^-1^), and kd the dissociation rate constant (AB → A + B, s^-1^).

### Reporting summary

Further information on research design is available in the Nature Portfolio Reporting Summary linked to this article.

## Supplementary information

Supplementary Information

Reporting summary

## Data Availability

All data to reproduce the findings reported here are available from: https://zenodo.org/records/14771076.

## code availability

The source code for EvoBind2 is available from https://github.com/patrickbryant1/EvoBind There is also a Google Colab that enables running EvoBind through a web interface: https://colab.research.google.com/github/patrickbryant1/EvoBind/blob/master/EvoBind.ipynb.
