# Supplementary material for: Design of linear and cyclic peptide binders from protein sequence information
Source: Commun Chem. 2025 Jul 22;8:211. doi: 10.1038/s42004-025-01601-3 (PMC12280060; doi:10.1038/s42004-025-01601-3)
Supplement: Supplementary file 1 — Supplementary Information [file 42004_2025_1601_MOESM1_ESM.pdf]

Supplementary information

Supplementary data

Supplementary Table 1. Linear binder design selection. One sequence was selected per length according to the lowest combined loss. The ‘length’ column represents the length of the selected peptide sequences. ‘afm_plddt’ and ‘evo_plddt’ are the AlphaFold-multimer and EvoBind2 plDDT (per-residue model confidence) scores respectively, which are measures of the predicted accuracy of the protein structure. ‘afm_loss’ (equation 2) and ‘evo_loss’ (equation 1) are the loss values associated with the AFM and EvoBind2 predictions respectively. The ‘sequence’ column contains the amino acid sequence of the protein. ‘%charged’ and ‘%hydro’ show the percentage of charged and hydrophobic amino acids in the sequence, respectively. The ‘combined_loss’ (sum of equations 1 and 2) is a combined measure of loss, considering both the AFM and EvoBind2 predictions. The kinetic properties of all the sequences towards the receptor protein were measured in the wet lab using Biacore 8K SPR and data are listed in the ‘Kd’ column. The PAE is the predicted aligned error from AFM and the Purity, the measured purity of the peptide according to LC-MS.

length	afm_plddt	afm_loss	evo_plddt	evo_loss	sequence	%charged	%hydro	combined_loss	Kd (M)	PAE	Purity (%)
8	97.0	0.00303	95.0	0.03621	GCRSCRSR	0.375	0.000	0.03923	0	3.10899	93.6
9	92.6	0.00039	91.1	0.03888	TMRDLQEPT	0.333	0.222	0.03927	0	3.42366	98.0
10	97.7	0.00055	96.1	0.03754	RSMADLDRTE	0.500	0.200	0.03809	0	3.56351	93.0
11	94.5	0.00251	91.6	0.03940	GAKYPRACRSM	0.273	0.182	0.04191	1.06E-06	3.611423	90.8
12	95.8	0.00473	92.8	0.03973	TARTIQKMDGHT	0.333	0.167	0.04446	8.54E-07	3.85132	90.6
13	95.2	0.00307	93.8	0.03920	GRELRPTCYPVGR	0.308	0.231	0.04227	4.79E-06	3.39942	90.9
14	94.8	0.00428	92.1	0.04213	GNERHLSETWPRNA	0.357	0.143	0.04641	0	3.37567	90.3
15	95.4	0.00212	92.4	0.04121	GRYPREVERTENPGA	0.400	0.133	0.04333	7.51E-09	3.505667	91.0
16	95.4	0.00186	89.5	0.04405	RDIREISSRGAENING	0.375	0.188	0.04591	2.56E-08	3.45376	93.5
17	96.4	0.00530	95.8	0.04247	WKLRDANCERAATNVCV	0.294	0.235	0.04776	0	3.34174	93.0

18	94.2	0.00854	94.0	0.04121	RNVQCQDECEDS LTNMAK	0.333	0.167	0.04975	6.82E-04	3.374 22	92.3
19	88.2	0.00550	93.6	0.04138	EKIMKGMRSEGD TNESDHD	0.526	0.158	0.04688	3.11E-04	3.717 99	95.3
20	85.8	0.00482	87.5	0.04830	GRGAPHITDRHEN MESVPAG	0.350	0.150	0.05311	0	9.268 26	90.6

Supplementary Table 2. Adversarial linear design selection.

One sequence was selected per length according to having an AFM loss >1 and lowest evobind loss. The 'length' column represents the length of the adversarial linear peptide sequences. 'afm_plddt' and 'evo_plddt' are the AlphaFold-multimer and EvoBind2 plDDT (per-residue model confidence) scores respectively, which are measures of the predicted accuracy of the protein structure. 'afm_loss' (equation 2) and 'evo_loss' (equation 1) are the loss values associated with the AFM and EvoBind2 predictions respectively. The 'sequence' column contains the amino acid sequence of the protein. '%charged' and '%hydro' show the percentage of charged and hydrophobic amino acids in the sequence respectively. The 'combined_loss' (sum of equations 1 and 2) is a combined measure of loss, considering both the AFM and EvoBind2 predictions. The kinetic properties of all the sequences towards the receptor protein were measured in the wet lab using Biacore 8K SPR and data are listed in the 'Kd' column. The PAE is the predicted aligned error from AFM and the Purity, the measured purity of the peptide according to LC-MS.

length	afm_plddt	afm_loss	evo_plddt	evo_loss	sequence	%charged	%hydro	combined_loss	Kd (M)	PAE	Purity (%)
8	39.6	1.03485	84.0	0.04595	LTGARRHP	0.375	0.125	1.08080	0	7.64731	91.0
9	34.8	1.20923	72.2	0.05115	YQPSRSDTH	0.333	0.111	1.26038	0	8.999545	93.9
10	30.2	1.26260	90.3	0.04549	HVRGCKSSPD	0.400	0.100	1.30809	0	3.77053	96.0
11	36.6	1.03688	93.6	0.03946	GAKYPIACRSH	0.273	0.182	1.07634	5.94E-06	10.00875	93.2
12	46.2	1.32138	77.7	0.05701	ARQMCSVDHKRS	0.417	0.167	1.37839	0	3.86608	96.0
13	32.9	1.22040	90.2	0.04111	WGPTRHMSGRCSE	0.308	0.154	1.26151	0	4.93705	97.2
14	66.1	1.42210	93.1	0.04315	WNNSTNRQVKDRCC	0.286	0.143	1.46526	3.42E-07	4.016189	95.1
15	33.0	1.06856	78.0	0.06513	ELKPCSCMEAKRPGH	0.400	0.133	1.13369	0	5.310634	91.5
16	31.1	1.32252	52.5	0.10206	RDHKEISPRGANNING	0.375	0.125	1.42459	0	4.51619	91.1
17	39.1	1.50744	93.4	0.04091	IRFEGCTGAGRGCGDRD	0.353	0.118	1.54834	0	3.693156	96.6
18	70.1	1.88598	90.6	0.04495	RNVHCENECSKSLTEMKK	0.444	0.167	1.93094	0	7.71942	94.9
19	29.9	1.06091	79.2	0.05648	PYNPNCCQRDMSDAQRDGM	0.263	0.158	1.11739	0	9.91427	97.8
20	34.8	1.11798	78.4	0.05579	GAGAPHISDRHENMESEPAG	0.350	0.100	1.17377	0	11.5399	97.3

Supplementary Table 3. Cyclic binder design selection. One sequence was selected per length according to the lowest combined loss. The 'length' column represents the length of the cyclic peptide sequences. 'afm_plddt' and 'evo_plddt' are the AlphaFold-multimer and EvoBind2 plDDT (per-residue model confidence) scores respectively, which are measures of the predicted accuracy of the protein structure. 'afm_loss' (equation 2) and 'evo_loss' (equation 1) are the loss values associated with the AFM and EvoBind2 predictions respectively. The 'sequence' column contains the amino acid sequence of the protein. '%charged' and '%hydro' show the percentage of charged and hydrophobic amino acids in the sequence respectively. The 'combined_loss' (sum of equations 1 and 2) is a combined measure of loss, considering both the AFM and EvoBind2 predictions. The lengths selected for experimental validation (top 4) are coloured green for the ones that were evaluated. Length 17 could not be synthesised by GenScript and is therefore coloured magenta. The remaining peptides were not evaluated experimentally. The PAE is the predicted aligned error from AFM and the Purity, the measured purity of the peptide according to LC-MS.

length	afm_plddt	afm_loss	evo_plddt	evo_loss	sequence	%charged	%hydro	combined_loss	Kd (M)	PAE	Purity (%)
8	95.21	0.00618	92.4	0.04303	PDERCNGV	0.38	0.13	0.04921		3.37246	
9	95.62	0.00447	94.5	0.04678	TTLDKRCPN	0.33	0.11	0.05125		3.38143	
10	92.32	0.00416	87.1	0.04456	EARDRMQVEG	0.50	0.20	0.04873	6.48E-06	3.72673	94.4
11	94.27	0.00450	95.3	0.04431	VEGNCRSPRMC	0.27	0.18	0.04881	0	3.36632	90.1
12	89.80	0.00420	94.9	0.04565	PNDRMCSSDSCE	0.33	0.08	0.04984		3.68063	
13	95.82	0.00296	96.2	0.04517	NSDSNTRCIECNH	0.31	0.08	0.04813	0	3.53968	91.5
14	96.51	0.00403	95.5	0.04183	TWMDADGSDSEGNN	0.29	0.14	0.04586	0	3.24551	97.7
15	94.68	0.00672	95.4	0.04380	REINNGGGYPHDSNS	0.27	0.13	0.05052		3.53343	
16	90.47	0.00693	93.9	0.04235	HDRSVSDGNEAEMEGR	0.50	0.13	0.04928		3.65643	
17	90.43	0.00127	94.1	0.04602	TCYFKYDHGDCNDFGPN	0.29	0.24	0.04729		3.72927	
18	85.53	0.01544	82.3	0.05251	TKVIGVNGQHFPKTDHGA	0.28	0.22	0.06795		4.28522	

19	69.38	0.02489	60.0	0.07747	TVELRFT HHPEALH AGEEN	0.42	0.21	0.10236		5.91 312	
20	91.52	0.00714	89.3	0.04975	DENLPRN HPQSDNT VEVGID	0.35	0.20	0.05689		3.71 765	

Supplementary figures

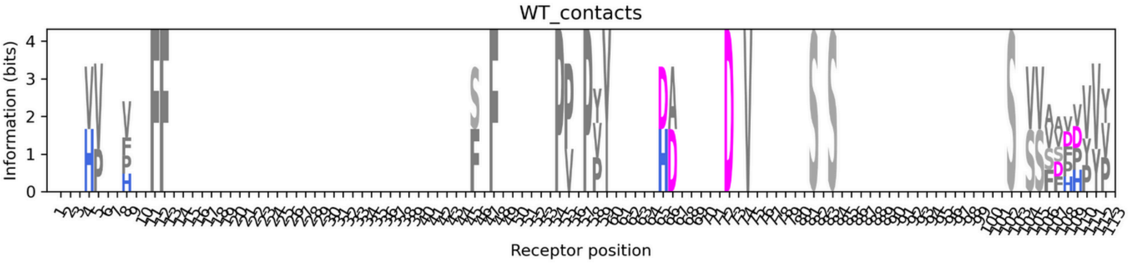

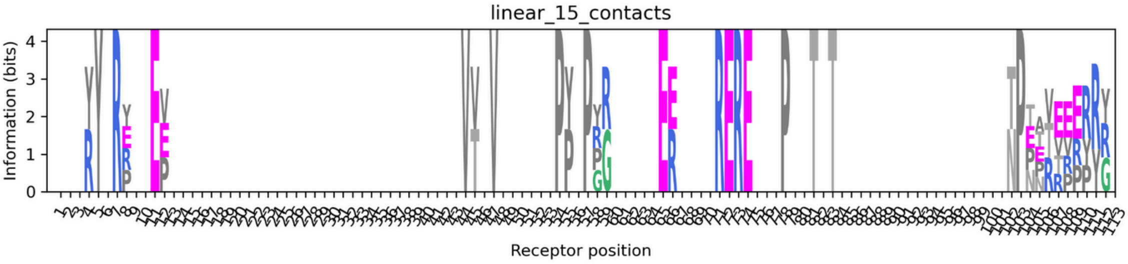

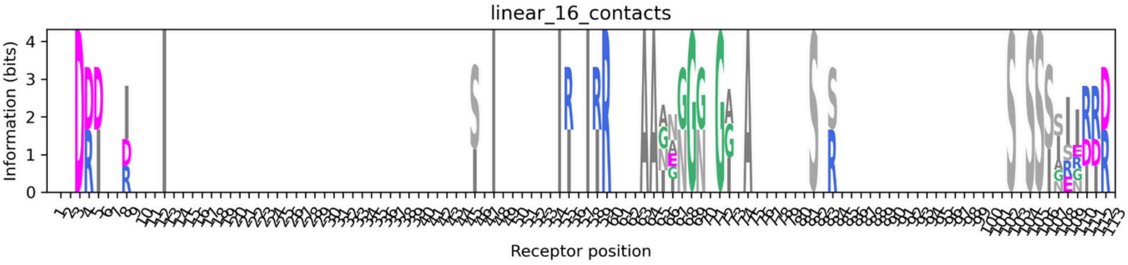

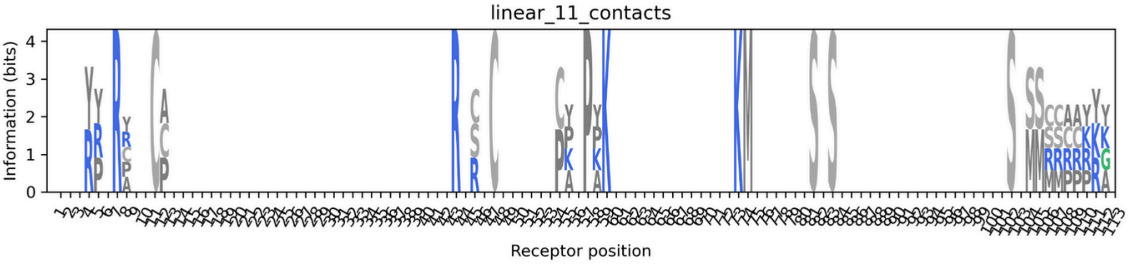

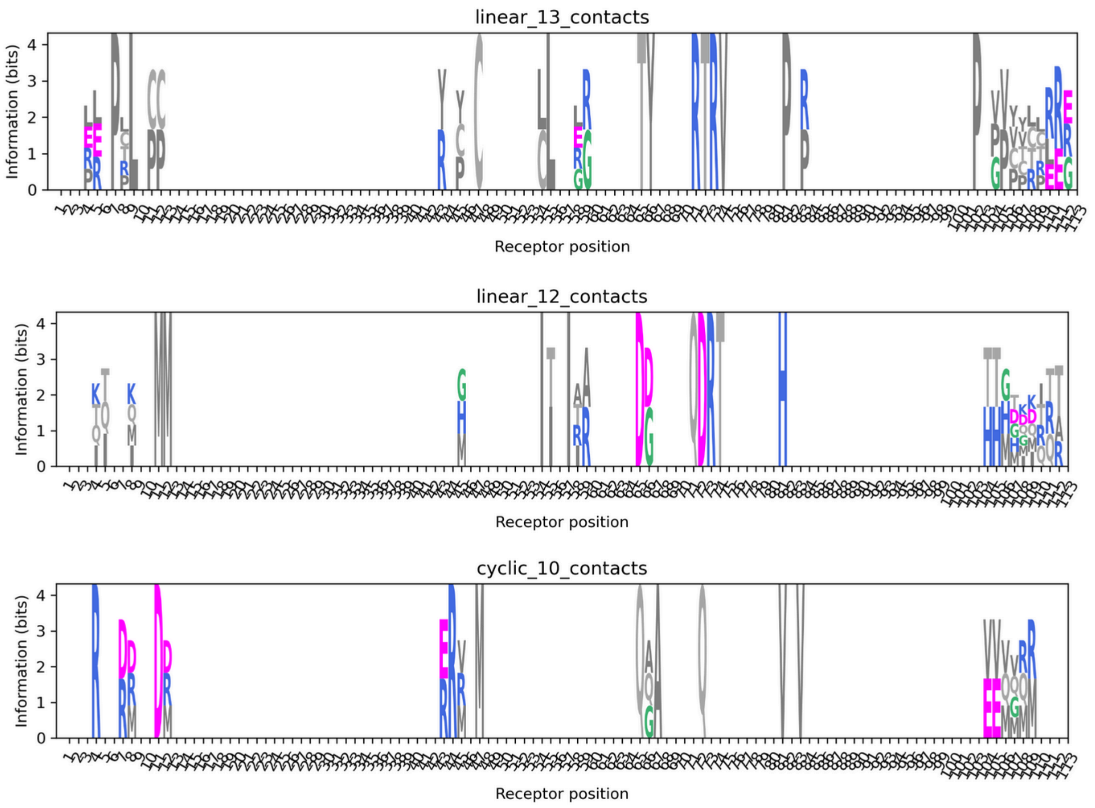

Supplementary Figure 1. Amino acid contacts of the binders for each position in the receptor compared with the WT. The number of contacts are 15, 10, 11, 7, 6 and 5 and the fractions of the number of WT contacts (65) are 0.23, 0.15, 0.32, 0.11, 0.09 and 0.8 for linear lengths 15, 16, 11, 13, 12 and cyclic 10, respectively.

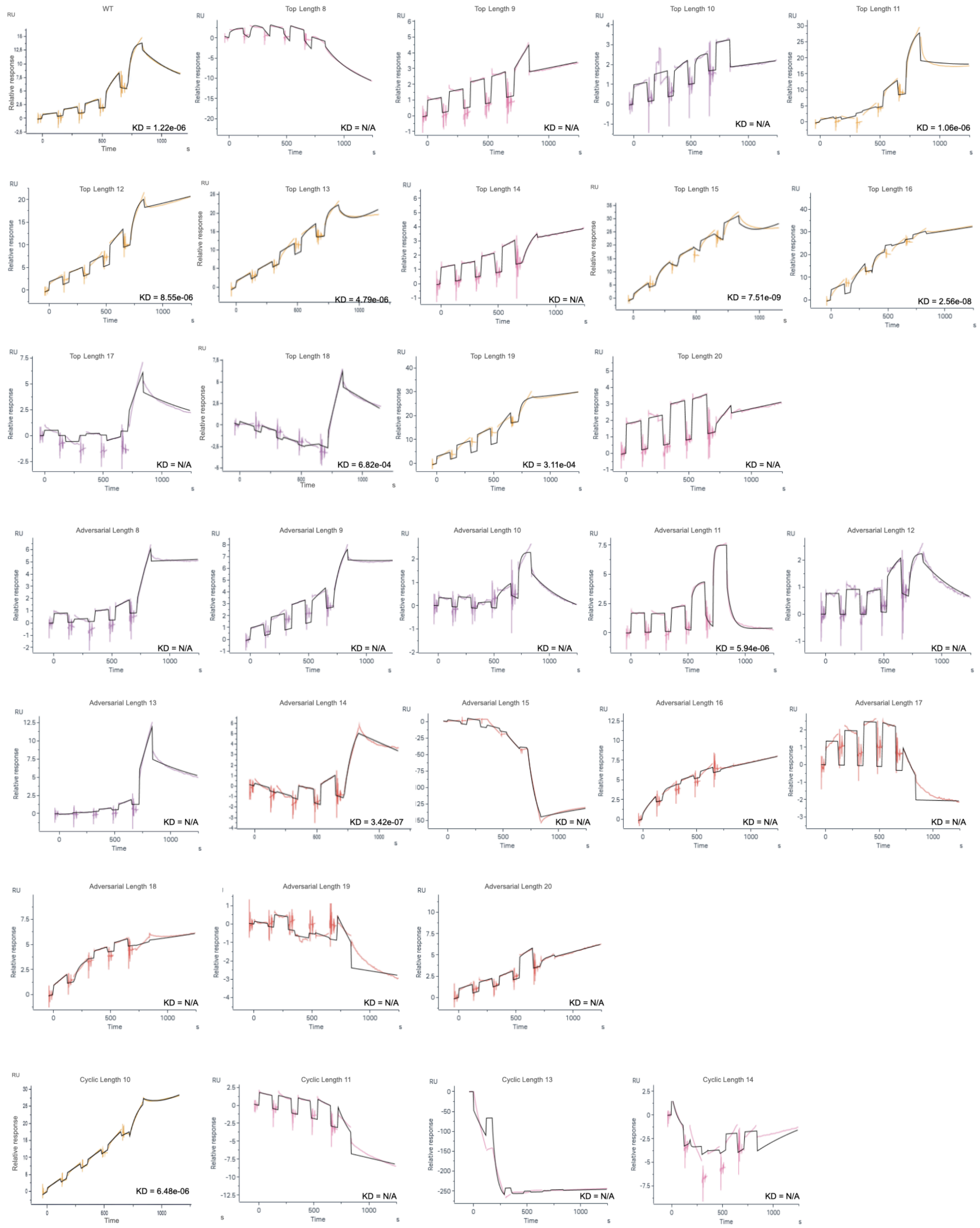

Supplementary Figure 2. Sensorgrams for all evaluated peptides. SPR sensorgrams obtained using Biacore 8K. The graphs show real-time binding interactions between immobilized ligands and analytes in solution. Each subfigure represents a separate binding experiment, displaying the response units (RU) on the y-axis versus time (seconds) on the x-axis. The colored lines represent the experimental data, while the black lines show the fitted curves based on a 1:1 binding model (Methods).

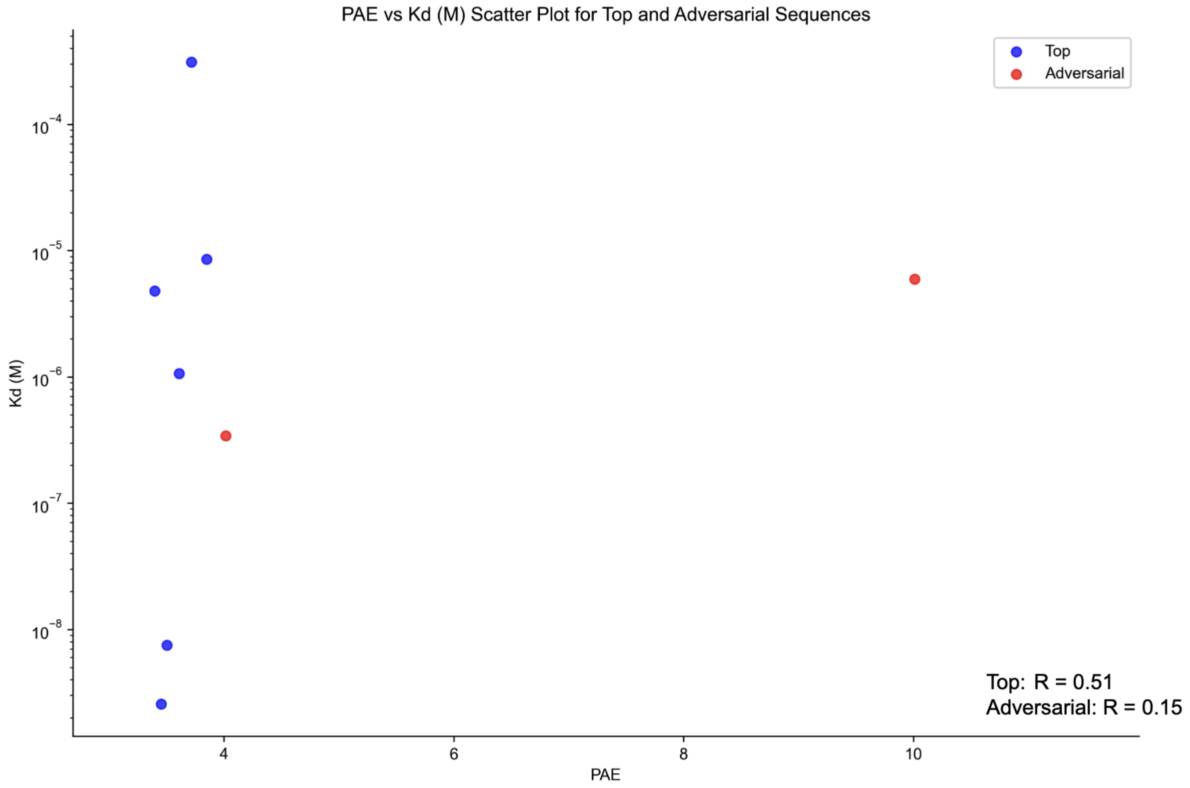

Supplementary Figure 3. PAE vs Kd. This scatter plot compares the Predicted Aligned Error (PAE) against the dissociation constant (Kd) for designed linear top selected and adversarial peptide binders. Blue points represent top designs, which cluster at lower PAE values (3-4) and red points show adversarial designs. One of the adversarial designs has low PAE and is a binder, and one has high but is binding as well. The Spearman correlations (R) are low compared to e.g. the AFM loss (Figure 4).

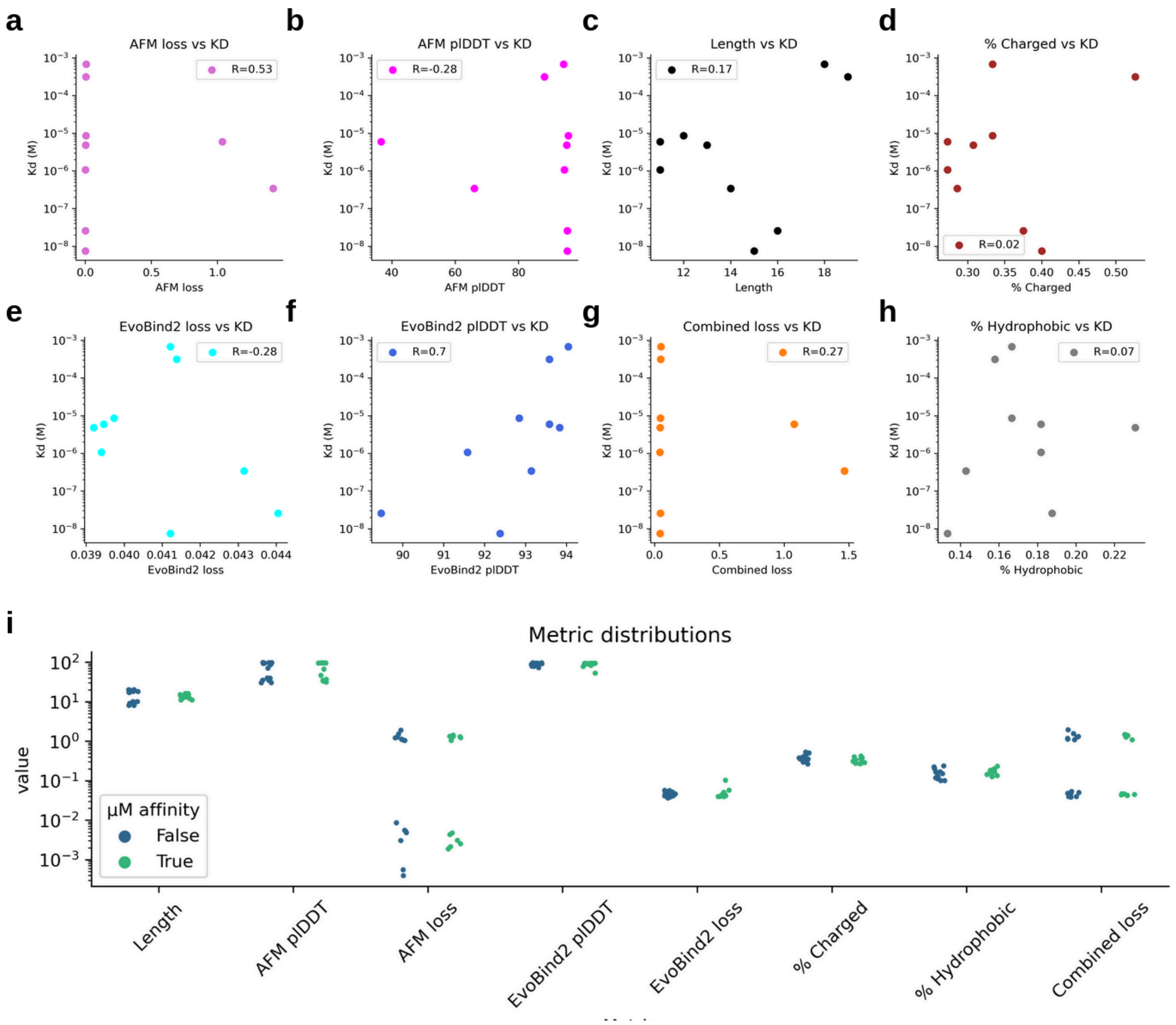

Supplementary Figure 4. a-h Scatter plots showing the affinities of linear peptides (KD) vs *in silico* metrics for designs with measurable affinity from the top and adversarial selection (n=9). The Spearman correlation coefficient (R) is annotated in each subfigure. Both the AFM loss and the EvoBind2 plDDT display a meaningful correlation with the measured Kd (R=0.96 and 0.79, respectively). **a)** AlphaFold-multimer loss (equation 2) **b)** plDDT from AlphaFold-multimer **c)** the length of designed peptide **d)** the fraction of charged amino acids in the sequence **e)** plDDT from EvoBind2 **f)** the loss from EvoBind2 (equation 1) **g)** the combined loss from EvoBind2 and AlphaFold-multimer (equations 1 and 2) **h)** the fraction of hydrophobic amino acids in the sequence. **i)** Strip plot of the categories in a-h divided by if µM affinity could be measured/not (n=26). No *in silico* metric separates true from false binders.

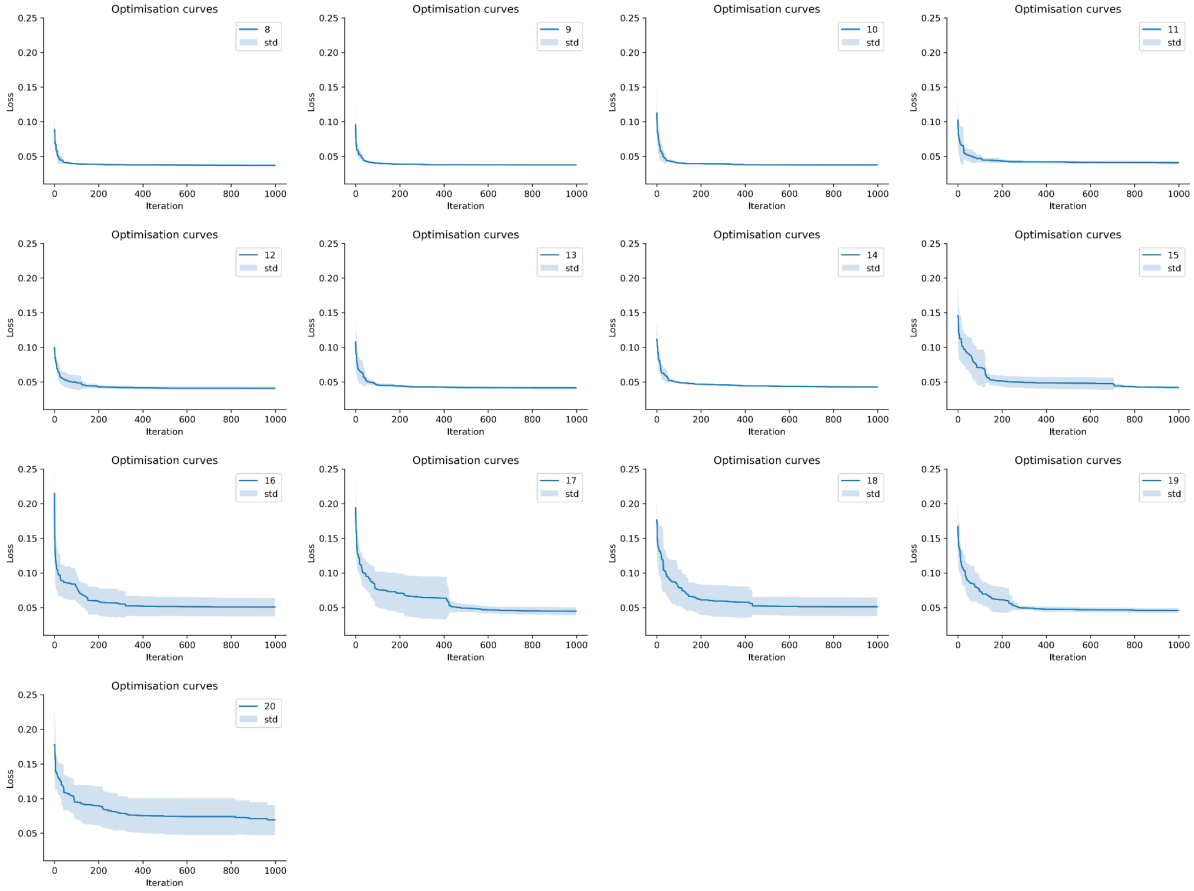

Supplementary Figure 5. Linear optimisation. EvoBind2 loss (equation 1) vs. Iteration curve showing the optimisation process 1000 iterations. The blue line represents the mean loss value for each peptide length across five different initialisations, and the shaded region is the standard deviation. The standard deviation increases with the length, suggesting the higher number of possibilities accompanied by a longer sequence length making it less likely to end up in a favourable design.

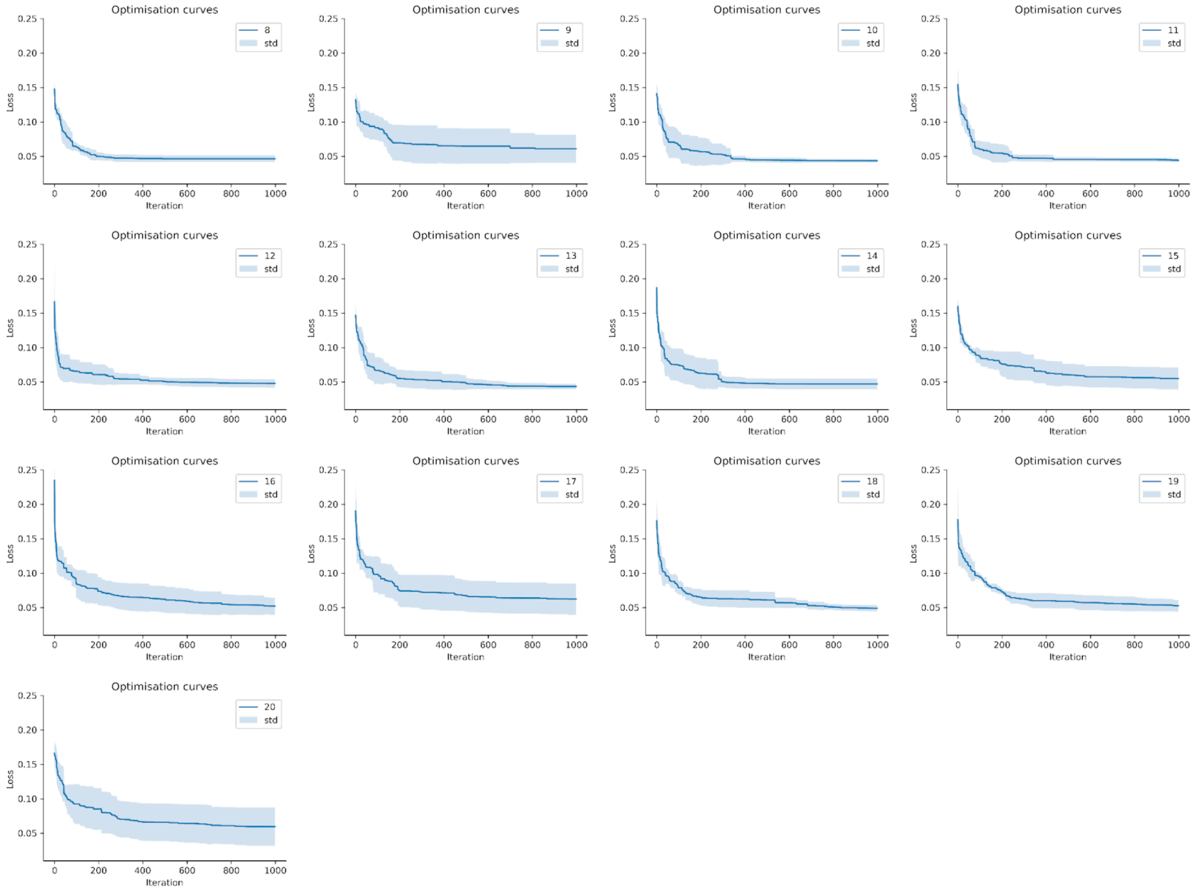

Supplementary Figure 6. Cyclic optimisation. EvoBind2 loss (equation 1) vs. Iteration curve showing the optimisation process 1000 iterations. The blue line represents the mean loss value for each peptide length across five different initialisations, and the shaded region is the standard deviation. The standard deviation does not increase with the length as with the linear selection.

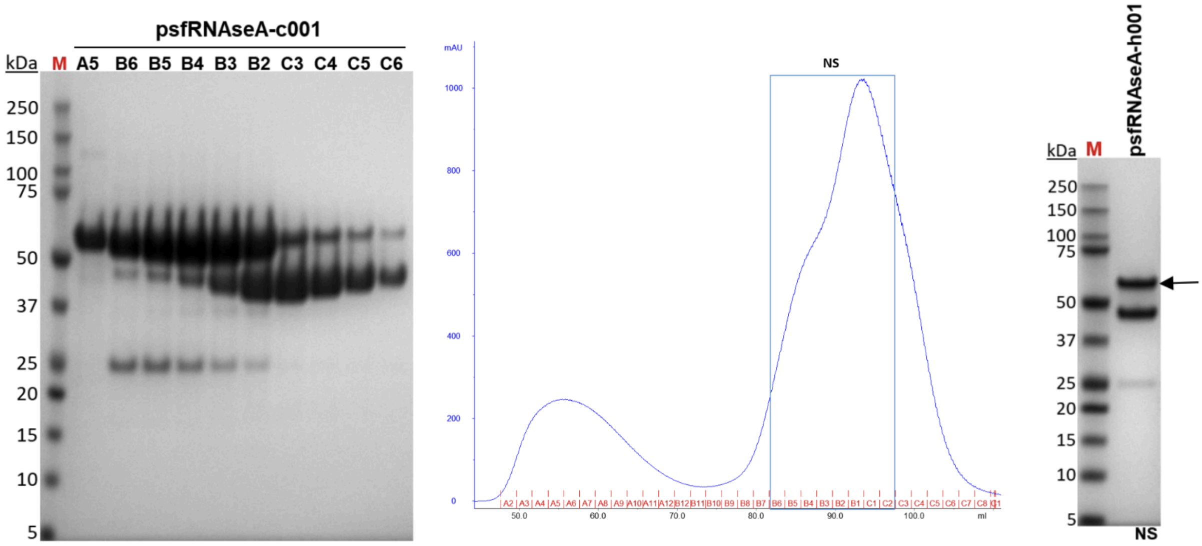

Supplementary Figure 7. MBP-8xHis-tag 1SSC Gel filtration elution profile and SDS-PAGE analysis. Batch purity was analysed using SDS-PAGE. For the SDS-PAGE, ~4

µg of protein was loaded. Protein concentration was calculated from UV absorbance at 280 nm using a theoretical extinction coefficient. Pooled psfRNAseA-h001 (NS) fractions: B6-B2.
